# Impact of a Workshop on Rural Healthcare Delivery on Medical Student Attitudes

**DOI:** 10.13023/jah.0704.06

**Published:** 2025-12-01

**Authors:** Robert L. Snedegar, Ryan Hayes, Jun Xiang, Treah Haggerty, Kendra Unger, Jason Oreskovich, Amie M. Ashcraft

**Affiliations:** West Virginia University; West Virginia University; West Virginia University; West Virginia University

**Keywords:** Appalachia, cross-sectional study, education, medical, rural population, undergraduate

## Abstract

**Introduction:**

Rural residents in the United States (U.S.) experience significant health disparities due to social determinants such as poverty, geographic isolation, and limited healthcare access. Physician shortages in rural areas further exacerbate these challenges.

**Purpose:**

This study evaluates the impact of an educational workshop on medical students’ attitudes toward rural healthcare barriers, aiming to determine whether targeted education can enhance their understanding and interest in rural practice.

**Methods:**

Third-year medical students at the West Virginia University School of Medicine participated in a workshop that included a 20-minute pre-recorded lecture and a 90-minute interactive virtual session. Pre- and post-surveys assessed students’ attitudes toward rural healthcare challenges. Responses were analyzed using paired t-tests with Bonferroni correction (p < 0.0017), stratified by rural background.

**Results:**

Of 97 students who completed the baseline survey, 67 completed both pre- and post-surveys (69% completion rate). After applying Bonferroni correction, four significant changes emerged: students reported greater confidence in addressing rural patient concerns (4.07 v 2.99, p < 0.0001), increased support for exempting rural patients from strict appointment timing rules (4.15 v 3.42, p < 0.0001), greater flexibility regarding follow-up scheduling (2.30 v 2.84, p < 0.0001), and enhanced recognition of financial strain from travel to Morgantown (4.70 v 4.43, p < 0.0001). Students from non-rural backgrounds demonstrated the most pronounced improvements in understanding rural-specific healthcare barriers.

**Implications:**

The workshop effectively improved medical students’ understanding of rural healthcare barriers, with particularly strong effects among students lacking prior rural exposure. These findings suggest that integrating structured educational experiences into medical curricula may enhance students’ preparedness for rural practice and help address physician shortages in underserved areas. Future research should explore the long-term impact of such interventions on career choices.

## INTRODUCTION

Rural residents in the United States (U.S.) exhibit significantly higher rates of adverse health outcomes than urban populations due to complex social determinants, including poverty, lower education levels, geographic isolation, and limited healthcare access.^1^–^3^ Compounding these challenges is the persistent shortage of physicians in rural areas, driven by physicians’ concerns about professional development, compensation, workload, and access to medical specialists.^4^–^5^

Physicians with rural backgrounds or exposure to rural health care during medical education are more likely to subsequently practice in these settings.^6^,^7^ Strategies such as financial incentives, specialized training, and community-based recruitment efforts have been implemented to address shortages, but innovative educational approaches are needed to prepare future physicians for rural practice.^8^–^12^ This study evaluates the impact of an educational workshop on medical students’ attitudes toward rural healthcare barriers, filling a gap in existing research.

This study examined whether a brief educational workshop could improve medical students’ understanding of the unique barriers to health care in rural communities. By targeting attitudes early in training, the workshop aimed to increase awareness, empathy, and openness to rural practice as a future career path.

## METHODS

### Workshop Components and Content

The educational workshop included a 20-minute pre-recorded lecture and a 90-minute interactive virtual session. The workshop was designed using established pedagogical frameworks for adult learning and professional development.^13^,^14^ The workshop content was developed collaboratively by members of the research team in partnership with rural patients and family members from local West Virginia (WV) communities who volunteered to share their healthcare experiences on camera. The pedagogical approach incorporated multiple evidence-based educational strategies, including pre-learning through recorded lectures, case-based learning through authentic patient narratives,^15^ facilitated small-group discussions,^16^ and simulation-based practice through mock patient scenarios.^17^

The workshop was developed specifically for third-year medical students during their Family Medicine clerkship rotation, representing a critical period for attitude formation about different practice settings. The intervention has been implemented and evaluated only in this single institutional setting. To assess the workshop’s impact, students completed pre- and post-surveys, requiring approximately 155 minutes in total.

### Participants

Participants were third-year medical students at the West Virginia University (WVU) School of Medicine, with protected time allocated during their Family Medicine Clerkship. The workshop was conducted in partnership with the WVU Center for Simulation Training and Education for Patient Safety (WV STEPS). Participation in the workshop learning activities was required as part of the Family Medicine curriculum. However, completion of the evaluation surveys was voluntary, and students would participate in the workshop without completing the research components.

### Survey Instrument

A custom survey instrument was developed incorporating both validated items from previous rural medical education research and newly created items specific to the workshop’s objectives. Approximately 18% of survey items were adapted from validated instruments: demographic questions from Royston and colleagues^18^ and rural practice belief items from Walker and colleagues.^19^ The remaining 82% were developed specifically for this workshop evaluation to assesses self-efficacy in addressing rural healthcare barriers, sensitivity to rural patient needs, and knowledge of rural health challenges.

Items not derived from previously validated instruments underwent content validation through expert review by the research team based in part on input from rural patients and family members during workshop development. The pre-workshop survey contained 42 questions, and the post-workshop survey contained 52 questions, with both using 5-point Likert scales (1 = disagree completely to 5 = agree completely).

Internal consistency reliability was assessed using Cronbach’s alpha (baseline α = 0.44, post-intervention α = 0.67). These moderate values reflect the multidimensional nature of the instrument, which was designed as a program evaluation tool across diverse domains rather than a validated scale for a single construct.

### Statistical Analysis

All tests in the current study were conducted using SAS (version 9.4, 2013, SAS Institute Inc. Cary, NC). Descriptive statistics were calculated for all survey items. Independent sample t-tests were conducted to evaluate the difference in baseline scores between participants from rural and non-rural areas. We used matched pair t-tests to determine if there was a significant difference between pre- and post-test scores of Likert scale survey questions. All tests were two-sided, and *p*-values of less than 0.05 were considered statistically significant.

This study was reviewed and deemed Not Human Subject Research (NHSR) by the West Virginia University Institutional Review Board (IRB) (protocol # 2108393436).

## RESULTS

Of the 97 third-year medical students who completed the baseline survey, 47.4% grew up in rural communities and 46.4% in non-rural communities (Table 1). Baseline comparisons revealed significant differences between rural and non-rural students on five survey items (Table 2). A total of 67 students (69%) completed both surveys and comprised the final sample, including 35 from rural backgrounds and 27 from non-rural backgrounds. Five students completed both surveys but had missing rural/non-rural background information and were excluded from stratified analyses. No significant differences in background were observed between survey completers and non-completers (*p* = 0.34).

To address concerns about multiple hypothesis testing, we applied a Bonferroni correction, setting the significance threshold at *p* < 0.0017. After applying this correction, four key findings were statistically significant (Table 3). Across all participants, students reported feeling significantly more prepared to address rural patients’ concerns after the workshop (4.07 v 2.99, *p* < 0.0001). Students also showed significantly greater agreement that rural patients should be exempt from a 20-minute rule for medical appointments (4.15 v 3.42, *p* < 0.0001) and were significantly less likely to agree that rural patients should follow up as regularly as local patients (2.30 v 2.84, *p* < 0.0001). Additionally, students showed significantly increased recognition of financial strain associated with traveling to Morgantown for medical care (4.70 v 4.43, *p* < 0.0001).

When examining stratified results by rural background, students from non-rural backgrounds showed the most robust significant changes. This group showed significant improvement in understanding follow-up scheduling flexibility for rural patients (mean change = −0.78, *p* < 0.0001) and increased recognition of financial strain associated with traveling to Morgantown for medical care (mean change = 0.26, *p* < 0.0001).

Several additional findings approached statistical significance but did not reach our adjusted significance threshold. Students showed trends toward increased support for telemedicine as an effective tool for rural follow-up care (*p* = 0.005), greater endorsement of coordinated same-day appointments (*p* = 0.002), and increased recognition of testing coordination needs for rural patients (*p* = 0.008). Among students from rural backgrounds, there were trends toward greater recognition that rural areas lack adequate recreational facilities (*p* = 0.006) and healthy grocery access (*p* = 0.03). Two items showed minimal non-significant changes in concerns about rural practice difficulty and social isolation (*p* = 0.88 and *p* = 0.80, respectively).

This pattern of results suggests that the workshop was effective in improving students’ understanding of rural healthcare barriers, with the most consistent effects observed in areas related to scheduling flexibility, patient preparation confidence, and recognition of logistical challenges faced by rural patients. The differential response patterns between rural and non-rural students indicate that prior rural exposure may influence how students process and integrate educational content about rural healthcare challenges, with students lacking rural experience showing the most pronounced improvements in understanding rural-specific healthcare barriers.

## IMPLICATIONS

This study is among the first to assess the impact of an undergraduate educational intervention on medical student attitudes toward rural healthcare delivery. The workshop’s combination of authentic patient narratives with interactive simulation provides a novel approach to addressing rural health education gaps during clinical training. While prior research has largely centered on graduate medical education, this study addresses a gap in understanding how early exposure may shape students’ perspectives on rural practice. Our findings suggest that even brief, structured educational experiences can meaningfully influence students’ awareness of rural health disparities.

The workshop significantly enhanced students’ understanding of logistical and systemic barriers faced by rural patients, including transportation challenges, financial strain, and limited access to coordinated care. Notably, students expressed increased confidence in addressing rural patients’ concerns and showed increased support for telemedicine and adaptive scheduling practices tailored to rural populations.^20^,^21^ The finding that non-rural students demonstrated the most pronounced improvements suggests that educational interventions may be particularly valuable for broadening the rural-prepared physician pipeline beyond tradition rural recruitment strategies focused primarily on students with existing rural connections.

Limitations include the single-institution design at a medical school with an established rural health mission, which may have introduced acquiescence bias.^22^,^23^ The immediate post-intervention survey may have led to recency bias,^24^,^25^ and the survey length could have contributed to respondent fatigue.^26^,^27^ The generalizability of findings may be limited by the focus on third-year students during Family Medicine clerkship and the specific Appalachian rural context, which may not represent rural healthcare challenges in other geographic regions or institutional settings.

The moderate internal consistency reliability (baseline α = 0.44, post-intervention α = 0.67) reflects the multidimensional nature of our instrument and represents a limitation for measuring attitude change, as the survey was designed as a program evaluation tool across several domains rather than a validated scale for a single construct. The 69% completion rate raises potential selection concerns, though no significant differences in rural background were observed between survey completers and non-completers.

Despite the limitations, these findings may have important implications for medical education and rural workforce development. The workshop’s brief format and minimal resource requirements make it feasible for integration into existing medical school curricula without substantial additional infrastructure. Integrating structured rural health content early in medical training may foster greater interest in rural practice and improve students’ preparedness to address rural health challenges. Future research should investigate whether such interventions have a lasting impact on students’ residency choices and eventual practice locations. Longitudinal studies and studies involving medical specialists could further inform strategies to strengthen the rural healthcare workforce.

**SUMMARY BOX**

**What is already known about this topic?**

Rural residents face significant healthcare access challenges, and medical students with early rural exposure are more likely to consider rural practice.

**What is added by this report?**

This study demonstrates that a brief, structured workshop significantly improved medical students’ understanding of rural health care barriers, with four key areas showing robust statistical significance after Bonferroni correction: patient preparation confidence, scheduling flexibility, appointment timing policies, and recognition of financial travel burdens. Students from non-rural backgrounds showed the most pronounced improvements, suggesting educational interventions may be particularly effective for broadening the rural prepared physician pipeline beyond students with existing rural connections.

**What are the implications for future research?**

Further research should examine whether early educational interventions influence long-term career decisions, whether effects differ by baseline rural exposure, and how to optimize brief interventions for maximum impact on attitudes and eventual practice location choices.

## Figures and Tables

**Table 1:** Characteristics of Third-Year Medical Students Completing Workshop During Family Medicine Clerkship (n = 97)

Survey Item		n (%)
**What would you consider the community that you grew up in?**		
	Rural	46 (47.4)
	Non-rural	45 (46.4)
	Unsure	6 (6.2)
**What would you consider the community that your spouse grew up in?**		
	N/A	53 (54.6)
	Rural	20 (20.6)
	Non-rural	22 (22.7)
	Unsure	2 (2.1)
**How many of your parents grew up in a rural area?**		
	0	27 (29.3)
	1	24 (26.1)
	2	41 (44.6)
**How many of your spouses’ parents grew up in a rural area?**		
	0	26 (45.6)
	1	10 (17.6)
	2	21 (36.8)

**Table 2:** Baseline Differences in Medical Student Participants by Rural Status (n = 91)

Survey Item	Rural Status		*p*-value†
	Rural (n=46) Mean (SD)	Non-rural (n=45) Mean (SD)	
**Followup1**: Rural patients should be expected to follow-up just as regularly as local patients.	2.63 (1.00)	3.13 (1.01)	0.02*
**Followup3**: Rural patient’s follow-up appointments should be coordinated to all be on the same day, if possible.	4.76 (0.43)	4.47 (0.66)	0.01*
**Travel3**: Patients expect more in-depth care when traveling long distances for care when compared to others.	4.20 (0.86)	3.82 (0.83)	0.04*
**Life4**: Social support in rural areas is limited compared with urban areas.	3.91 (1.01)	3.42 (1.10)	0.03*
**Employ8**: I will practice in a rural area after completion of my residency.	3.41 (0.86)	2.84 (0.82)	0.002*

NOTES:

† *p*-value from independent samples t-test

* *p* <0.05

**Table 3:** Differences in Survey Questions Before and After RHCD Workshop by Rural Status

	Rural (n=35)		Non-rural (n=27)		All (n=67)†	
Survey Question	Mean (SD)	*p*	Mean (SD)	*p*	Mean (SD)	*p*
**Initial evaluation of self and system questions**						
*Self1:* Residents of rural counties have equal access to healthcare as their urban counterparts.	0.03 (0.82)	0.84	0.19 (1.18)	0.42	0.07 (0.96)	0.53
*Self2:* I do a good job of taking extra time to address all of a rural patient’s concerns and ask about barriers to testing.	0.14 (1.00)	0.41	−0.04 (0.85)	0.82	0.06 (0.90)	0.59
*Self3:* Medical providers do a good job of addressing the concerns of rural patients who come to Morgantown for care.	−0.09 (0.70)	0.47	0.04 (0.85)	0.82	−0.04 (0.75)	0.63
*Self4:* I do a good job of taking extra time to address all of rural patient’s concerns and ask about barriers to testing.	0.23 (0.91)	0.15	0.19 (0.79)	0.23	0.22 (0.83)	0.04
*Self5:* I feel prepared to address all of the concerns of visiting rural patients when they are seeking care here in Morgantown.	1.03 (1.04)	<0.0001*	1.11 (1.05)	<0.0001*	1.09 (1.06)	<0.0001*
*Self6:* It is the physician’s job to ensure “all of the loops” are closed and rural patients can get the tests they require for care and will be able to attend follow-up appointments.	0.06 (1.03)	0.74	0.07 (0.92)	0.68	0.07 (0.94)	0.52
**Late to appointment questions**						
*Late1:* Rural patients should be exempt from a “20-minute rule” on appointments (if they arrive more than 20 minutes late, they will not be permitted to attend their appointment).	0.89 (0.93)	<0.0001*	0.52 (0.89)	0.006	0.73 (0.90)	<0.0001
**Testing sensitivity questions**						
*Test1:* Rural patients should be able to have all of their tests done in Morgantown on the same day as their appointments.	0.11 (0.58)	0.25	0.33 (0.73)	0.03	0.22 (0.67)	0.008
*Test2:* Rural patients should be asked about where they want to have their testing performed.	0.09 (0.56)	0.37	0.11 (0.42)	0.18	0.10 (0.50)	0.09
**Follow-up questions**						
*Follow-up1:* Rural patients should be expected to follow-up just as regularly as local patients.	−0.40 (1.09)	0.04	−0.78 (0.89)	0.0001*	−0.54 (0.99)	<0.0001*
*Follow-up2*: Telemedicine can be an effective tool for follow-up appointments.	0.31 (0.47)	0.0004*	0.15 (0.82)	0.36	0.22 (0.62)	0.005
*Follow-up3*: Rural patient’s follow-up appointments should be coordinated to all be on the same day if at all possible.	0.11 (0.47)	0.16	0.30 (0.67)	0.03	0.24 (0.61)	0.002
**Morgantown travel questions**						
*Travel1*: Traveling to Morgantown is a barrier to seeking health care.	0.11 (0.53)	0.21	0.15 (0.53)	0.16	0.16 (0.54)	0.02
*Travel2*: Attending an appointment in Morgantown is a significant financial strain for rural patients.	0.23 (0.55)	0.02	0.26 (0.66)	0.05	0.27 (0.59)	0.0004*
*Travel3*: Patients expect more in depth care when traveling long distances for care when compared to others.	0.17 (0.82)	0.23	0.15 (0.86)	0.38	0.15 (0.82)	0.14
**Rural access to positive lifestyle changes questions**						
*Life1*: Rural patients should be able to adhere to lifestyle changes just as easily as urban patients.	−0.09 (1.17)	0.67	0 (1.07)	1.00	−0.10 (1.10)	0.44
*Life2*: There are poor recreational facilities and in rural areas.	0.46 (1.09)	0.02	0.30 (1.07)	0.16	0.37 (1.08)	0.006
*Life3*: There is limited access to healthy groceries in rural areas.	0.37 (0.84)	0.01	−0.04 (0.98)	0.85	0.24 (0.91)	0.03
*Life4*: Social support in rural areas is limited compared with urban areas.	0 (1.16)	1.00	0.33 (1.52)	0.26	0.16 (1.29)	0.30
**Rural employment assessment questions**						
*Employ1*: I believe that there are good opportunities for employment in rural areas.	−0.11 (1.02)	0.51	−0.33 (1.03)	0.11	−0.19 (1.00)	0.12
*Employ2*: I believe that there are good opportunities for career advancement in rural areas.	0 (0.97)	1.00	0.48 (1.05)	0.03	0.16 (1.01)	0.19
*Employ3*: I believe that professional isolation is a problem when working in rural areas.	−0.14 (0.88)	0.34	0.33 (0.96)	0.08	0.09 (0.95)	0.44
*Employ4*: I believe that rural areas have insufficient recreational facilities.	0.26 (0.92)	0.11	0.30 (1.14)	0.19	0.25 (0.99)	0.03
*Employ5:* I believe that rural areas have good social support.	−0.09 (0.85)	0.56	−0.04 (1.13)	0.87	−0.09 (0.97)	0.45
*Employ6:* I believe that rural practice is too hard.	0.09 (0.82)	0.54	−0.04 (0.85)	0.82	0.01 (0.83)	0.88
*Employ7:* I believe that working in rural areas means being too isolated from friends.	0.20 (1.05)	0.27	−0.04 (0.76)	0.80	0.03 (0.95)	0.80
*Employ8*: I will practice in a rural area after completion of my residency.	0 (0.59)	1.00	0.04 (0.76)	0.80	0.06 (0.67)	0.47
*Employ9*: Rural physicians have greater community impact than non-rural physicians.	0.14 (0.88)	0.34	0.11 (0.75)	0.45	0.18 (0.83)	0.08
*Employ10*: Rural physicians have less prestige than other physicians.	−0.29 (1.07)	0.12	−0.11 (1.01)	0.57	−0.21 (1.04)	0.10
*Employ11*: I believe that working in rural areas provides more opportunity to practice a variety of skills.	0.14 (0.81)	0.30	0.22 (0.70)	0.11	0.16 (0.75)	0.08

Notes: *p*-values from paired t test or Wilcoxon signed rank test,

* *p* <0.0017

† 5 students completed both surveys but did not indicate rural vs non-rural background and were excluded from stratified analysis.

## References

[1] Douthit N Kiv S Dwolatzky T Biswas S Exposing some important barriers to health care access in the rural USA Public Health 2015 129 6 611 20 Available from: 10.1016/j.puhe.2015.04.001 26025176

[2] Long AS Hanlon AL Pellegrin KL Socioeconomic variables explain rural disparities in US mortality rates: Implications for rural health research and policy SSM Popul Health 2018 6 72 4 30225336 10.1016/j.ssmph.2018.08.009 PMC6138992

[3] Nelson WA Pomerantz A Howard K Bushy A Medical ethics in rural healthcare Am J Bioeth 2010 10 12 1 3 10.1136/jme.2006.015966 PMC2598268 17329381

[4] Coughlin SS Clary C Johnson JA Berman A Heboyan V Benevides T Continuing challenges in rural health in the United States J Environ Health Sci 2019 5 2 90 2 32104722 PMC7043306

[5] Rural Health Information Hub Healthcare access in rural communities [Internet] Rural Health Information Hub 2024 [cited 2025 Jan 8]. Available from: https://www.ruralhealthinfo.org/topics/healthcare-access

[6] Strasser R Learning medicine in rural settings: A global perspective Int J Med Educ 2016 7 304 6

[7] Sahu PC Chintaram F Sahu AC Medical student’s attitude towards serving rural areas: A cross-sectional study in Maharashtra, India Panacea J Med Sci 2022 12 387 92

[8] Arredondo K Touchett HN Khan S Current programs and incentives to overcome rural physician shortages in the United States: A narrative review J Gen Intern Med 2023 38 Suppl 3 916 22 10.1007/s11606-023-08122-6 37340266 PMC10356718

[9] Eidson-Ton WS Rainwater J Hilty D Henderson S Hancock C Nation CL Training medical students for rural, underserved areas: a rural medical education program in California J Health Care Poor Underserved 2016 27 4 1674 88 27818431 10.1353/hpu.2016.0155

[10] Meade ZS Doobay-Persaud A Tseng J Demographics and medical school exposures to rural health influence future practice Surgery 2022 172 6 1665 72 36127171 10.1016/j.surg.2022.08.016

[11] Goodfellow A Ulloa JG Dowling PT Talamantes E Chheda S Bone C Predictors of primary care physician practice location in underserved urban and rural areas in the United States: A systematic literature review Acad Med 2016 91 9 1313 27119328 10.1097/ACM.0000000000001203 PMC5007145

[12] Pfarrwaller E Sommer J Chung C Maisonneuve H Nendaz M Junod Perron N Impact of interventions to increase the proportion of medical students choosing a primary care career: A systematic review J Gen Intern Med 2015 30 9 1349 58 26173529 10.1007/s11606-015-3372-9 PMC4539313

[13] Kirkpatrick DL Kirkpatrick JD Evaluating training programs: The four levels 3rd ed San Francisco, CA Berrett-Koehler Publishers 2016

[14] Mezirow J Learning as transformation: Critical perspectives on a theory in progress Adult Education Quarterly 2000 50 1 3 12

[15] Thistlethwaite JE Davies D Ekeocha S The effectiveness of case-based learning in health professional education. A BEME systematic review: BEME Guide No. 23 Med Teach 2012 34 6 e421 44 22578051 10.3109/0142159X.2012.680939

[16] Edmunds S Brown G Effective small group learning: AMEE Guide No. 48 Med Teach 2010 32 9 715 26 20795801 10.3109/0142159X.2010.505454

[17] Motola I Devine LA Chung HS Sullivan JE Issenberg SB Simulation in healthcare education: a best evidence practical guide. AMEE Guide No. 82 Med Teach 2013 35 10 e1511 30 23941678 10.3109/0142159X.2013.818632

[18] Royston PJ Mathieson K Leafman J Ojan-Sheehan O Medical student characteristics predictive of intent for rural practice Rural Remote Health 2012 12 2 2107 Available from: http://www.rrh.org.au 22873948

[19] Walker JH DeWitt DE Pallant JF Cunningham CE Rural origin plus a rural clinical school placement is a significant predictor of medical students’ intentions to practice rurally: a multi-university study Rural Remote Health 2012 12 2 1908 Available from: http://www.rrh.org.au 22239835

[20] Chunchu K Mauksch L Charles C Ross V Pauwels J Telehealth interventions and outcomes across rural communities in the United States: Narrative review JMIR mHealth and uHealth 2021 9 8 e29575 10.2196/29575 PMC8430850 34435965

[21] National Rural Health Association The impact of telehealth policy on rural health access: Policy Brief November 2024 Available from: https://www.ruralhealthinfo.org/topics/telehealth-health-it

[22] Rammstedt B Goldberg LR Borg I The measurement equivalence of Big-Five factor markers for persons with different levels of education Journal of Research in Personality 2010 44 1 53 61 20401177 10.1016/j.jrp.2009.10.005 PMC2854544

[23] Billiet JB McClendon MJ Modeling acquiescence in measurement models for two balanced sets of items Structural Equation Modeling 2000 7 4 608 28

[24] Holbrook AL Krosnick JA Moore D Tourangeau R Response order effects in dichotomous categorical questions presented orally: The impact of question and respondent attributes Public Opinion Quarterly 2007 71 3 325 48

[25] Kowalski M Vaske JJ Measuring changes with traditional and retrospective pre-posttest self-report surveys for a brief intervention program Evaluation and Program Planning 2023 99 102283 10.1016/j.evalprogplan.2023.102323 37276793

[26] Ben-Nun P Respondent fatigue Lavrakas PJ Encyclopedia of Survey Research Methods Thousand Oaks, CA Sage Publications 2008 743

[27] Ghafourifard M Survey fatigue in questionnaire based research: The issues and solutions J Caring Sci 2024 13 4 214 15 39974826 10.34172/jcs.33287 PMC11833437

